# Melatonin: The potential avenues in dentistry

**DOI:** 10.12688/f1000research.159942.1

**Published:** 2025-01-14

**Authors:** Sanidhya S, Deepa Kamath, Aradhya Sinha, Diya Kamath

**Affiliations:** 1 Department of Periodontology, Manipal College of Dental Sciences, Managlore, Manipal Academy of Higher Education, Manipal, Karnataka, 575001, India; 2 BDS, Manipal College of Dental Sciences, Manipal, Karnataka, India

**Keywords:** periodontal disease, melatonin, melatonin for implant dentistry, melatonin for bone regeneration, osseointegration

## Abstract

Melatonin, the “sleep hormone,” shows significant promise in dentistry owing to its antioxidant, anti-inflammatory, and immunomodulatory properties. It is beneficial for treating periodontal disorders and aiding osseointegration of dental implants. Additionally, melatonin helps to manage dental anxiety, offering an alternative to traditional sedatives.

Periodontal disease is orchestrated by bacterial plaques along with an exaggerated immune-inflammatory host response. Treatment of periodontitis not only involves the removal of plaque, but also aims to minimize the cytokine load and control the reactive oxygen species burden in the tissues, which would re-establish a healthy periodontium and a balanced bone metabolism.

Melatonin is known to exert beneficial effects, such as regulation of circadian rhythm, bone remodeling, and antimicrobial effects.

Recent studies have demonstrated the successful use of melatonin as an adjunct to mechanical debridement for the treatment of periodontal disease. Its various uses include systemic administration of melatonin after one-stage full-mouth Non-Surgical Periodontal Therapy in healthy subjects as well as patients.

This article provides a summary of the various clinical applications of melatonin, describing its mechanism of action, uses, and potential avenues for future research in dentistry.

## Introduction

Melatonin, primarily known as the “sleep hormone” produced by the pineal gland, has shown promising applications in dentistry. Its antioxidant, anti-inflammatory, and immunomodulatory properties make it beneficial for treating dental diseases, such as periodontal disorders, and aid in the osseointegration of dental implants. Additionally, the anxiolytic effects of melatonin can help manage dental anxiety, offering a potential alternative to traditional sedatives.

Melatonin has shown potential for use in various dental fields. In endodontics, it is effective for direct pulp capping and pulp preservation because of its antioxidant properties, which protect the dental pulp cells, promote healing, and reduce inflammation. It also aids in pulp tissue regeneration by modulating immune responses and preserving tooth vitality. In orthodontics, the anti-inflammatory and bone metabolism modulation properties of melatonin enhance bone mineral density, minimize root resorption, and improve implant stability and tooth movement. This can lead to healthier bone and tissue responses during orthodontic treatments. Its anxiolytic and analgesic properties are beneficial for oral surgery, as they reduce preoperative anxiety and postoperative pain. Its anti-inflammatory and antioxidant effects promote healing and reduce the surgical stress. In prosthodontics, melatonin enhances osseointegration and bone regeneration around dental implants, thereby improving implant stability and success rates. Their antioxidant properties help extend the longevity and functionality of prosthetic devices. In pedodontics, melatonin’s anxiolytic and sedative effects manage dental anxiety in children, making them more cooperative during dental procedures. Its antioxidant properties support the overall health of young patients by reducing oxidative stress.

Periodontitis is a chronic infection/inflammatory illness caused by bacteria and is characterized by an increased loss of tooth-supporting tissues. Periodontitis is the most common cause of edentulism and tooth loss in adults worldwide. ^1^ It is also intimately connected to other systemic disorders, such as Alzheimer’s disease, cardiovascular disease, cancer, and diabetes, all of which have a significant effect on people’s quality of life. ^2^ Periodontitis, the most prevalent chronic inflammatory illness in humans, has significant socioeconomic and health implications. According to the first Global Burden of Illness Study, severe periodontitis is the sixth most common illness in humans, affecting 11.2% of the world’s population. ^3^

Page and Kornman established a classic model of the etiology of periodontal disease in 1997 by demonstrating the interaction between the host response and microbial challenge, which promotes the development of periodontitis, ^4^ and demonstrated for the first time that tissue destruction is induced by both the immune-inflammatory response and the indirect impacts of bacteria. Microbial dysbiosis and an imbalance in host reactions cause the development of periodontitis. Dental plaque biofilm production causes gingivitis by accelerating the creation of a dysbiotic environment and causing the host immune-inflammatory response to become dysregulated. The subsequent breakdown of periodontal tissues is caused by overproduction of inflammatory chemokines and cytokines, unbalanced bone metabolism, and an increase in reactive oxygen species (ROS). ^5^ The primary goals of periodontal treatment are to reduce the extreme immune-inflammatory response and restore a symbiotic interaction between the host and the bacteria.

Hence, periodontitis in the oral cavity has the potential to cause serious harm to both systemic and global health. Therefore, there is an urgent need to put more effort into treat and prevent periodontitis. To properly manage periodontitis, it is necessary to understand its etiology or the molecular pathways that give rise to this condition. ^6^

## The therapeutic ability of melatonin in the oral cavity ( Figure 1)

**Figure 1.**
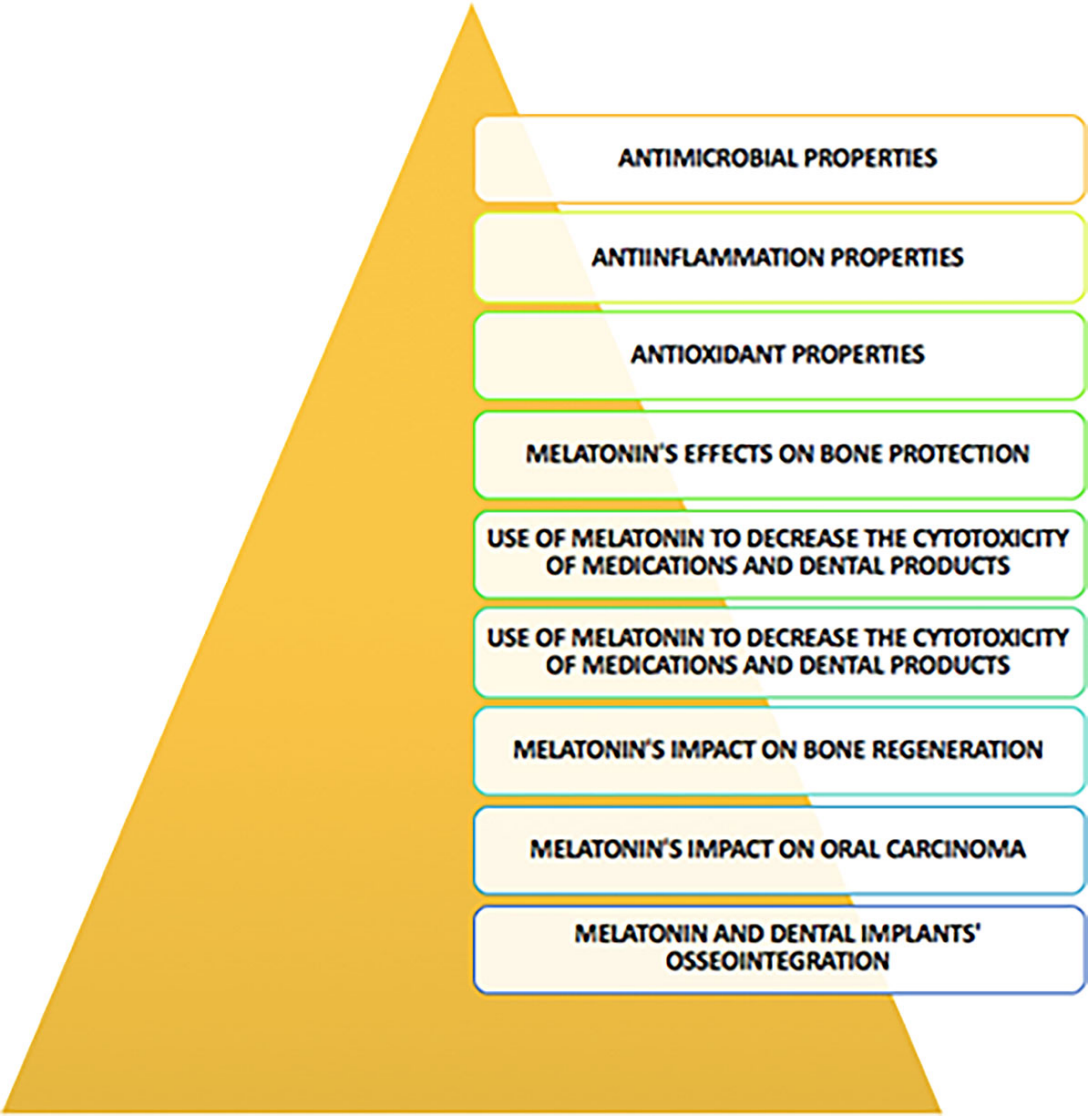
Therapeutic ability of melatonin in the oral cavity.

### Antimicrobial properties

The Antimicrobial activity of melatonin has been demonstrated against a variety of microorganisms, including viruses, fungi, bacteria, and parasites. Through a variety of ways, including the disruption of cell membranes, interference with DNA replication, and modulation of immune responses to improve the body’s ability to fight infections, it can directly restrict the development and replication of these pathogens. Furthermore, by lowering oxidative stress, which can impair microbial defenses, the antioxidant properties of melatonin enhance antibacterial effectiveness. Strong anti-infection characteristics were also observed for the natural hormone melatonin. in vitro studies have shown that melatonin can hinder the growth of *Acinetobacter baumannii,* methicillin-resistant *Staphylococcus aureus* and *Pseudomonas aeruginosa.* ^7^ Methicillin-resistant *Pseudomonas aeruginosa, Acinetobacter baumannii,* and *Staphylococcus aureus* have been demonstrated to be prevented by melatonin in in vitro experiments. The antibacterial effect of this hormone is often associated with immune responses that reduce the production of inflammatory cytokines and accelerate healing of bacterial lesions. For instance, melatonin and its receptor agonist ramelteon have antibacterial actions on planktonic-cultured *P. gingivalis.* Therefore they disrupt already-formed biofilms, prevent *P. gingivalis* biofilm growth, and lower *P. gingivalis* biofilm survival rates. It seems reasonable to assume that melatonin, which has potent antibacterial properties, also has anti-infection effects against oral pathogens, such as *Aggregatibacter actinomycetemcomitans* and *Tannerella forsythia.* ^8^ Further research is required to confirm these findings. Although studies in this field are still underway, melatonin appears to have potential as a natural remedy for preventing infections and boosting immune system function.

### Anti-inflammation properties

In addition to its role in sleep regulation, melatonin also has anti-inflammatory effects. By preventing the synthesis of pro-inflammatory cytokines and increasing the activity of anti-inflammatory molecules, it can modify a variety of immune responses and reduce inflammation. Melatonin also scavenges free radicals that fuel inflammation by acting as an antioxidant. Although encouraging, further studies are required to completely understand the scope and underlying mechanisms of the anti-inflammatory effects of melatonin.

Improved inflammation control may greatly lower the risk of tissue loss, because the abundance of inflammatory responses is a major cause of periodontal degeneration. Aspirin, NSAIDs, and corticosteroids are anti-inflammatory drugs that are known to cause serious side effects, such as bone issues. Melatonin has been shown to have powerful anti-inflammatory effects with no negative side effects. ^9^ This hormone can prevent periodontal tissue damage by regulating inflammatory reactions. Additionally, it showed elevated levels of pro-inflammatory cytokines, a high receptor activator of the nuclear factor kappa beta/Osteoprotegerin ratio, and TLR4/MyD88 activity. Notably, melatonin substantially reduced TLR4/MyD88-mediated pro-inflammatory cytokine production to restore normal RANKL/OPG signalling. Kara et al *.* showed that melatonin reduces periodontal tissue loss and inflammatory cytokines (IL-1 and TNF-) in rats with periodontitis. ^10^ Melatonin may also inhibit IL-1-stimulated TIMP-1, MMP-1, and CXCL-10 synthesis in human periodontal ligament cells.

### Antioxidant properties

Melatonin is believed to have been generated between 3.0 and 2.5 billion years ago to aid in the neutralisation of toxic O _2_ during photosynthesis by photosynthetic bacteria. ^11^ Although the original antioxidant function of melatonin has been preserved after more than three billion years of evolution, its other functions have significantly increased. Melatonin is widely acknowledged for its potent antioxidant and free radical-scavenging properties. Unlike most conventional antioxidants, melatonin metabolites can also neutralize oxygen derivatives. Because of this cascade reaction, this hormone is far more effective than other antioxidants, including glutathione, NADH, vitamin C, and vitamin E. ^12^

Much work has been undertaken to reduce extreme ROS in periodontal tissue as a result of improved understanding of ROS’s impact of ROS on tissue deterioration. Melatonin may be a good choice because it is the strongest antioxidant. According to a randomized controlled clinical study, melatonin considerably lowered the levels of MMP-9 in GCF and boosted antioxidant capacity (TAC). ^13^ In comparison with Non-Surgical Periodontal Therapy (NSPT) alone, NSPT combined with melatonin significantly reduced periodontal pocket depths in individuals with periodontitis and diabetes, according to a meta-analysis of two Randomized Controlled Trials. The delivery of DL-buthionine sulfoximine (BSO) and glutamate (GLUT) to Wistar rat gingival fibroblasts causes the creation of superoxide anions and cell loss, which are entirely stopped by melatonin. Additionally, it showed elevated levels of pro-inflammatory cytokines, a high receptor activator of the nuclear factor kappa beta/Osteoprotegerin ratio, and TLR4/MyD88 activity. Notably, melatonin substantially reduced TLR4/MyD88-mediated pro-inflammatory cytokine production to restore normal RANKL/OPG signalling. Melatonin may also inhibit IL-1-induced MMP-1,TIMP-1, and CXCL-10 synthesis in human periodontal ligament cells. ^14^

### Effects on bone protection

The most severe effects of periodontitis include bone resorption and tooth loss. Melatonin has been shown to have a positive effect on bone repair in various ways. For instance, melatonin accelerates the production and rate of type I collagen production in human bone cells and human osteoblastic cell lines. It also activates sirtuin 1, which stimulates osteogenic differentiation in bone marrow mesenchymal stem cells and inhibits adipogenesis while enhancing osteogenesis in human mesenchymal stem cells. ^15^ Additionally, melatonin inhibits RANKL-induced osteoclastogenesis, which prevents bone resorption. Additionally, melatonin may shield the bone in the mouth. Melatonin inhibits IL-1, RANK, and RANKL expression levels while increasing OPG expression levels in rats with investigational periapical lesions to exert bone-protective and anti-inflammatory effects. Melatonin also drastically lowered the bacterial localization scores in periodontal tissues. Melatonin can promote the osteogenic development of dental pulp mesenchymal stem cells (DPSCs), and DPSCs preconditioned with melatonin can repair bone lesions in vivo. Activation of myeloperoxidase and alveolar bone resorption, and statistically, melatonin reduced RANKL and osteoclast activity. In addition, it protects osteoblasts from drug-induced damage caused by drugs. For example, the use of chlorhexidine causes MC3T3 cells to have poor morphology, elevated levels of total ROS and superoxide, and decreased numbers of osteoblasts that are metabolically and physiologically active. ^16^ Notably, melatonin can mitigate the damage caused by chlorhexidine in MC3T3 cells, safeguarding osteoblasts during chlorhexidine treatment. Additionally, melatonin may be useful for the treatment of peri-implantitis. In the lipopolysaccharide-induced peri-implantitis rat model, melatonin reduced the incidence of peri-implantitis by lowering the number of osteoclasts, decreasing the generation of pro-inflammatory cytokines, and limiting harm to the alveolar bone. ^17^

### Impact on bone regeneration

Melatonin enhances osteoblast development and promotes bone formation, as is widely known. Melatonin has been shown to increase the expression of genes such as osteocalcin and alkaline phosphatase, which are involved in bone mineralization. This encourages the creation of structurally sound bone tissue by causing the accumulation of calcium and other minerals in the bone matrix. The immunomodulatory, antioxidant, and anti-inflammatory properties of melatonin may increase its osteoconductive effects. The cells responsible for bone resorption, osteoclasts, have been shown to be suppressed by melatonin. Melatonin inhibits osteoclast activity, which is essential for effective bone regeneration, maintaining bone mass, and preventing excessive bone loss.

Bone graft materials and bioactive scaffolds are utilized to promote the redevelopment of bone that has vanished because of periodontal infection or tooth loss. ^18^ When exposed to calcium aluminate scaffolds that also contain platelet-rich plasma and melatonin, human osteoblasts has been observed to multiply. Melatonin-based scaffolds have potential for guided tissue regeneration in the future.

### Melatonin and dental implants’ osseointegration

Osseointegration is the process by which a dental implant combines with the surrounding bone, and its influence on this process has been studied. Melatonin has been shown to facilitate angiogenesis and formation of new blood vessels, which are essential for supplying oxygen and nutrients to the implant site throughout the healing process. ^19^ Enhanced blood flow can facilitate osseointegration of dental implants and accelerate tissue recovery. Dental implants must have their surfaces altered before being placed into the periodontal bone to optimize their bioactivity. Ion leakage, leftover particles, and bioactive coating delamination are some of the drawbacks of modified implants. Unstable implant coatings can be successfully replaced by melatonin. Interthread bone development and mineralization were much higher at implant sites treated with melatonin powder than at the control implant sites. The osteogenic outcomes of porcine bone grafts in calcium-coated titanium dental implants were enhanced by melatonin. Moreover, the effects of melatonin on implant sites in vivo appeared to be enhanced when combined with growth hormone. Fibroblast growth factor-2 and melatonin injections have been found to increase the amount of bone in contact with zirconia and titanium implants. However, more extensive studies in both animals and humans are necessary to fully understand how melatonin affects the ability of implants to promote bone growth. According to Gomez-Moreno et al *.* (2015), the bone development surrounding implants benefits from the action of melatonin. ^20^

Additionally useful in the battle against peri-implantitis, melatonin suppresses the generation of pro-inflammatory cytokines, lowers production of osteoclasts thereby reducing bone resorption. ^21^ In vitro, melatonin stimulates osteoblastic growth and activity, while inhibiting osteoclastic formation and activity. Melatonin is also used in radiation therapy as it lowers oxidative stress in the hard and soft tissues of the oral cavity. It also increases the longevity of periodontal ligament cells by modulating autophagy. Furthermore, melatonin affects how The body reacts to stress, particularly the stress induced by surgical operations such as implant installation. Melatonin may help osseointegration indirectly by lowering stress-related variables that might obstruct the healing process. Recent studies have investigated the possible influence of melatonin on dental implant osseointegration, that is, the integration of the implant with the surrounding bone.

### Use of melatonin to decrease the cytotoxicity of medications and dental products

Several drugs affect the periodontium. Proksch et al. found that melatonin decreased the levels of reactive oxidant species and the cytotoxic effects of generally utilized oral antiseptic medicines such as chlorhexidine. ^22^ In addition, it shields periodontal cells from bisphosphonate necrosis. To avoid osteonecrosis of the jaw caused by bisphosphonates, topical melatonin therapy should be used before dental surgery, and methacrylate chemicals, which are known to have cytotoxic effects, are present in common dental polymers. Because of its antioxidant properties, melatonin has been proven to shield dental pulp cells, in contrast to DNA destruction caused by methacrylate. Thus, when used as gel mouthwashes, sublingual tablets, or gels, melatonin can reduce the genotoxic effects of dental materials created by methacrylates.

### Melatonin’s impact on oral carcinoma

Studies have been conducted on the effects of melatonin on oral carcinoma and mouth cancer. Although there is some evidence to support its potential benefits in the prevention and treatment of oral cancer, further research is necessary to fully understand its effects. Studies have shown that they possess anticancer properties, including pro-apoptotic, antimetastatic, and antiproliferative effects. These qualities can help stop the growth and metastasis of oral cancer cells. Since Reactive Oxygen Species are involved in the development of precancerous lesions, such as lichen planus and leukoplakia, the antioxidant properties of melatonin aid in the prevention or treatment of oral cancer. It can alter the immune system, boosting the activity of immune cells that are engaged in immunological defense and cancer surveillance. This immunosuppressive action could help the body to identify and eliminate oral cancer cells. Additionally, radiation therapy, which is frequently used to treat oral cancer, protects against melatonin-protected healthy tissues. Its radioprotective properties, when combined with radiation treatment, have been studied. This could increase the vulnerability of cancer cells to radiation-induced cell death while shielding healthy oral tissues from the harmful effects of radiation.

According to recent in vitro studies, melatonin may stop the progression of oral cancer by blocking the activation of metalloproteinase-9. Therefore, melatonin-containing toothpastes, gels, and rinses may aid in the prevention and slowing of oral cancers. ^15^

Therefore, further studies, including clinical trials, are required to completely clarify the effectiveness, appropriate dose, and safety profile of melatonin in the context of treating and preventing oral cancer, even though preliminary evidence points to a possible significance for the hormone in this regard. Melatonin should also be considered in conjunction with other proven treatments and lifestyle changes as a part of an all-encompassing strategy for the prevention and treatment of oral cancer.

## Effect of melatonin on periodontal therapy

According to a randomized clinical trial by Gonde et al *.* in 2022, Incorporating NSPT with an intrapocket injection of 1% melatonin gel for a week improves clinical and radiological results. ^1^ Tinto M et al. said in 2020 that using melatonin capsules for a month after NSPT caused a greater increase in Clinical Attachment Level (CAL) and a decrease in Probing Depth (PD). ^23^ The PD, CAL, and other important periodontal parameters might be greatly enhanced by adjunctive melatonin intake (topical and systemic). ^10^

The most popular approach for treating periodontitis is scaling and root planing (SRP), which involves mechanical removal of plaque. ^24^ The majority of patients’ periodontal conditions may improve after using these basic periodontal therapies. Some individuals still have increasing attachment loss after SRP, but plaque removal by itself is unable to stop the overwhelming inflammatory immune response and restore the microenvironment to normal. Moreover, due to the difference in the host’s genetic risk factors, systemic diseases, as well as the environmental and acquired risk factors, the extent of periodontal tissue loss and response to periodontal therapy fluctuate greatly across individuals. Therefore, it may be wiser to consider adjunctive therapies like host-modulation therapy as a viable option.

An endogenous hormone called “Melatonin” regulates the sleep-wake cycle and has a variety of biological impacts. Numerous biological effects of this compound have been identified, including regulation of infection, bone remodeling, circadian rhythm, antioxidant activity, and anti-inflammation. ^8^ The main areas of clinical investigation are the therapeutic advantages for immunological applications, cancer, neuroprotection, and problems with sleep and circadian rhythm. ^9^ With good outcomes from both laboratory and clinical investigations, interest in its use as a host-modulation medicine in the field of periodontology has increased.

Periodontitis can be treated with the beneficial effects of melatonin. In addition to Non-surgical periodontal treatment (NSPT), the local application of melatonin gel has been shown to improve clinical and radiographic results. ^25^ Comparing NSPT with placebo therapy to NSPT after one stage of full mouth, systemic melatonin delivery leads to a better clinical attachment level (CAL) increase and a decrease in probing depth (PD) decrease. Recent systematic reviews and meta-analyses of the provided data have concluded that supplemental melatonin administration (topically and systemically) can considerably enhance PD, CAL, and other important periodontal parameters. Additionally, the periodontal health and inflammatory and antioxidant parameters of patients with type 2 diabetes were improved by systemic melatonin administration. ^11^ Furthermore, it may have a beneficial effect on bone formation around implants in the field of implant dentistry, even though there is still a dearth of information, and more research is necessary to aid its clinical significance. In addition, according to a study by Srinath et al *.* in 2010, melatonin levels in salivary and GCF decreased in patients with periodontitis. ^12^

Although bacteria-induced infections can cause periodontitis, the severe immune-inflammatory response is mostly responsible for the destruction of the periodontal tissue. Overexpressed cytokines and chemokines (including IL-1, TNF, and MMPs), elevated ROS levels, a high receptor activator of nuclear factor kappa beta/osteoprotegerin ratio, and other factors contribute to inflammation. ^26^ Periodontopathogen-induced infection elimination is important for minimizing inflammatory cytokine expression levels, managing reactive oxygen species levels in periodontal tissue, and reestablishing normal bone metabolism to regulate periodontitis and diminish tissue breakdown. ^27^ Melatonin has a variety of biological properties that make it an effective periodontal therapy. ^28^

## Conclusion

The diverse properties of melatonin make it a valuable resource in dentistry. It has been shown to have beneficial outcomes when used as an adjunct to scaling and root planing to treat periodontal disease. Its antioxidant, anti-inflammatory, and immune-modulating effects enhance treatments across various dental fields, from endodontics and orthodontics to oral surgery and prosthodontics. In addition, its anxiolytic properties make it particularly useful in pedodontics, ensuring a more comfortable experience for young patients. As research continues, its role in dental practice can expand, offering new avenues for improved patient care and outcomes. Further studies should aim to standardize the treatment protocols using melatonin in terms of dosage, frequency of use, and therapy time. However, Melatonin, as an adjunct to scaling and root planning, offers a potential treatment option with a wide range of benefits for periodontal therapy.

## Ethics and consent

No ethics and consent were required.

## Data Availability

No data are associated with this article.
