# Supplementary material for: Melatonin: The potential avenues in dentistry
Source: F1000Res. 2025 Jan 14;14:77. [Version 1] doi: 10.12688/f1000research.159942.1 (PMC11926526; doi:10.12688/f1000research.159942.1)
Supplement: Peer review history

# Reviewer report: Reviewer response for version 1

**DOI:** 10.5256/f1000research.175736.r371153

**Published:** 2025-03-20

**Authors:** Biju Thomas

**Affiliations:** 1 AB Shetty Memorial Institute of Dental Sciences, NITTE (Deemed to be University), AB Shetty Memorial Institute of Dental Sciences, Mangaluru, India

Summary and Relevance

 This article reports on melatonin which is of interest in dentistry today due to its multiple properties like antimicrobial, anti inflammatory, antioxidant and immunomodulation. The article highlights the possibility of melatonin playing a role in management of periodontal diseases and also possibly enhancing the osseointegration of dental implants ,which is making rapid strides as a treatment modality for replacement of missing teeth.

 Evidence based literature is available today on the multiple beneficial effects of melatonin and hence this article is relevant as a review on the current status of melatonin and its potential benefits in periodontal and implant therapy.

 Strengths and Weaknesses

 The strength of this article is its narration of the therapeutic benefits of melatonin, a hormone which is ignited interest in the field of dentistry , in the oral cavity and the advantages of its application in a variety of oral diseases including oral carcinoma.

 The weakness of this article is that it is just a narrative review  and hence no conclusion can be drawn from it, only some possibilities and future prospects can be stated. Hence it is only of limited interest.

Is the review written in accessible language?

Yes

Are all factual statements correct and adequately supported by citations?

Yes

Are the conclusions drawn appropriate in the context of the current research literature?

Yes

Is the topic of the review discussed comprehensively in the context of the current literature?

Yes

Reviewer Expertise:

Periodontitis , stem cells , etc

I confirm that I have read this submission and believe that I have an appropriate level of expertise to confirm that it is of an acceptable scientific standard.

# Reviewer report: Reviewer response for version 1

**DOI:** 10.5256/f1000research.175736.r371151

**Published:** 2025-03-19

**Authors:** Anupama Rao

**Affiliations:** 1 Yenepoya Dental college, Mangalore, India

It is very well written review article on Melatonin - the potential avenues in dentistry. author has done extensive search on previous literature and very well compiled its finding in the present article. limitation of previous studies to identify the standard Therapeutic dosage in periodontal therapy, recommendation regarding one time or multiple administration in pocket (local drug delivery) , long term evaluation of clinical results can be incorporated in discussion part. overall its a well compiled review in my opinion.

Is the review written in accessible language?

Yes

Are all factual statements correct and adequately supported by citations?

Yes

Are the conclusions drawn appropriate in the context of the current research literature?

Yes

Is the topic of the review discussed comprehensively in the context of the current literature?

Yes

Reviewer Expertise:

local drug delivery, herbs in periodontal therapy

I confirm that I have read this submission and believe that I have an appropriate level of expertise to confirm that it is of an acceptable scientific standard.

# Reviewer report: Reviewer response for version 1

**DOI:** 10.5256/f1000research.175736.r371147

**Published:** 2025-03-18

**Authors:** Gustavo Fernandes

**Affiliations:** 1 Periodontics and Oral Medicine Department, A. T. Still University - Missouri School of Dentistry & Oral Health, St. Louis, Missouri, USA

Dear authors,

 This article is a review; it is titled "Melatonin: The potential avenues in dentistry". It is a very simple review that included a limited number of references; this type of article brings bias in its development

 Title: include the type of article in the title (narrative review)

 Reference : around 56% of the refs. were published in the last 5 years. I recommend to increase a little it (for around 70%, observed the type of article). Update them, please.

 - For the subtopic "Effects on bone protection", I recommend to include the recent article: [Ref 2]

 - for the topic "Effect of melatonin on periodontal therapy", I suggest to include the recent article below.

 (new ref.) [Ref 1]

 - See other articles to update the refs., please.

Is the review written in accessible language?

Yes

Are all factual statements correct and adequately supported by citations?

Yes

Are the conclusions drawn appropriate in the context of the current research literature?

Yes

Is the topic of the review discussed comprehensively in the context of the current literature?

Yes

Reviewer Expertise:

Periodontics, Biomaterials, Implantology, Oral Surgery, Rehabilitation

I confirm that I have read this submission and believe that I have an appropriate level of expertise to confirm that it is of an acceptable scientific standard, however I have significant reservations, as outlined above.

## References

[1] : Effects of Melatonin in the Non-Surgical Treatment of Periodontitis: A Systematic Review. *Applied Sciences* .2022;12(22) : 10.3390/app122211698 10.3390/app122211698

[2] : Use of Local Melatonin with Xenogeneic Bone Graft to Treat Critical-Size Bone Defects in Rats with Osteoporosis: A Randomized Study. *J Funct Biomater* .2024;15(5) : 10.3390/jfb15050124 10.3390/jfb15050124 38786635 PMC11122056
